# Nutraceutical formulations and natural compounds for the management of chronic diseases

**DOI:** 10.3389/fnut.2025.1682590

**Published:** 2025-10-15

**Authors:** Ghizal Fatima, Sadaf Khan, Vani Shukla, Wajdy Awaida, Duo Li, Yulia Sh Gushchina

**Affiliations:** ^1^Department of Biotechnology, Era University, Lucknow, India; ^2^Department of Food and Nutrition, Era University, Lucknow, India; ^3^Department of Biology and Biotechnology, Faculty of Science, American University of Madaba, Amman, Jordan; ^4^Institute of Nutrition and Health, Qingdao University, Qingdao, China; ^5^Department of General and Clinical Pharmacology, Medical Institute, People’s Friendship University of Russia, Moscow, Russia

**Keywords:** chronic diseases, nutraceuticals, natural compounds, antioxidant, therapeutic potential, functional foods, bioactive compounds, metabolic disorders

## Abstract

Chronic non-communicable diseases including cancer, diabetes, cardiovascular, neurodegenerative, and autoimmune disorders pose mounting global health and economic challenges. Conventional drugs often focus on symptom management, frequently accompanied by side effects and rarely reversing disease progression. Nutraceuticals bioactive compounds sourced from foods, herbs, and marine organisms, offer a promising alternative due to their inherent anti-inflammatory, antioxidant, immunomodulatory, neuroprotective, and cardioprotective properties. This review synthesizes current evidence on key nutraceutical classes (e.g., polyphenols, flavonoids, omega-3 fatty acids, probiotics, plant alkaloids), elucidating their molecular mechanisms such as oxidative stress mitigation, immune modulation, gene regulation, and signaling pathway interactions and highlighting therapeutic applications across major chronic conditions. Addressing a critical limitation, we analyze advanced delivery technologies (e.g., nano-formulations, encapsulation, liposomes, micro- and hydrogels, co-administered bioenhancers) designed to enhance bioavailability and targeting. We also discuss navigating hurdles such as regulatory inconsistencies, safety concerns, herb–drug interactions, and the need for standardization. To fully incorporate nutraceuticals into modern healthcare, the review emphasizes the imperative for rigorous clinical validation, manufacturing quality control, and long-term safety monitoring. Finally, we propose future directions including personalized nutraceutical strategies, AI-assisted discovery, and global regulatory harmonization positioning nutraceuticals as sustainable and evidence-based adjuncts or alternatives in chronic disease management.

## 1 Introduction

Non-communicable diseases (NCDs), another name for chronic diseases, are long-term ailments that often develop gradually and last a person’s entire life. These encompass a wide range of illnesses, including musculoskeletal disorders like arthritis, diabetes mellitus, cancer, chronic respiratory diseases, and cardiovascular diseases (such as hypertension and coronary artery disease). The World Health Organization (1) estimates that chronic diseases account for around 74% of deaths worldwide each year, with cardiovascular diseases alone causing more than 17.9 million deaths. These disorders are frequently caused by a confluence of behavioral, physiological, environmental, and genetic variables, and their prevalence is increasing as a result of aging populations, poor diet, stress, and sedentary lifestyles (133). Although there is no denying that traditional pharmaceutical therapies have increased life expectancy and quality of life, they are not without drawbacks. Instead than addressing the underlying causes of chronic diseases, many synthetic medications concentrate on symptom suppression. Furthermore, side effects, drug resistance, and decreased patient compliance are often linked to prolonged prescription usage. In patients with arthritis, for example, long-term use of nonsteroidal anti-inflammatory medicines (NSAIDs) may result in renal impairment, cardiovascular risks, and stomach ulcers. Similar to this, even if they are effective, several chemotherapeutic medicines used to treat cancer can have crippling side effects that drastically lower quality of life (2).

Given these drawbacks, natural substances and nutraceuticals are gaining popularity throughout the world as potential supplements or substitutes for traditional treatments. Nutraceuticals, which are food-derived products that offer health or medicinal advantages beyond simple nourishment, contain a variety of bioactive substances, including vitamins, minerals, polyphenols, flavonoids, omega-3 fatty acids, and plant extracts. Among other things, these compounds have been demonstrated to have cardioprotective, anti-inflammatory, antioxidant, and anti-cancer properties (3). Because of their natural origin, multiple methods of action, and reduced toxicity, nutraceuticals are being more and more integrated into preventative and therapeutic healthcare. Systematic studies that compile the available data, examine bioavailability issues, and indicate potential future paths in the use of nutraceuticals for the treatment of chronic diseases are desperately needed as this field of study grows.

## 2 Aim of the review

This review systematically investigates the therapeutic potential of natural compounds and nutraceutical formulations in the prevention and management of chronic diseases. In light of the growing global burden of non-communicable diseases and the limitations of long-term pharmacotherapy, the review highlights how bioactive compounds derived from natural sources such as polyphenols, flavonoids, probiotics, and plant-based alkaloids may offer effective adjuncts or alternatives to conventional treatments. Emphasizing their antioxidant, anti-inflammatory, immunomodulatory, neuroprotective, and cardioprotective properties, the review explores the biological activities, mechanisms of action, and clinical efficacy of these nutraceuticals across a spectrum of chronic conditions, including cardiovascular diseases, diabetes, cancer, neurodegenerative disorders, and autoimmune diseases. A key focus is on overcoming challenges related to bioavailability and targeted delivery, through advanced formulation strategies such as nanoformulations, encapsulation, and synergistic combinations with other compounds or pharmaceuticals. The review also addresses regulatory frameworks, safety considerations, and standardization issues that currently hinder the broader clinical adoption of nutraceuticals. By synthesizing current scientific evidence and clinical applications, this review aims to guide healthcare professionals, researchers, and policymakers toward the informed integration of evidence-based nutraceuticals into mainstream healthcare, while identifying promising directions for future research.

## 3 Methodology

This narrative review is based on a comprehensive search of peer-reviewed literature published between 2000 and 2024 in PubMed, Scopus, ScienceDirect, and Google Scholar. Searches combined controlled vocabulary and free-text keywords using Boolean operators, with terms including “nutraceuticals,” “natural compounds,” “antioxidants,” “polyphenols,” “cancer prevention,” “cardiovascular health,” “diabetes management,” “neuroprotection,” “inflammation,” “autoimmune disorders,” and “nanoformulations.” The initial search yielded (insert number) records; after screening and eligibility assessment, (insert number) articles were included. Eligible studies comprised original research, reviews, meta-analyses, and clinical trials reporting mechanisms, therapeutic outcomes, safety, or formulation strategies. Non-English, unavailable full texts, unrelated topics, and commentaries were excluded. Compounds were selected based on frequency in literature, strength of evidence, and clinical relevance. Preference was given to publications from the last decade (2014–2024), with older landmark studies included when foundational.

## 4 Overview of nutraceuticals and natural compounds

Utilizing natural substances and nutraceuticals has become a viable strategy for managing chronic illnesses and preserving health in recent years. These compounds, which come from natural extracts, herbs, or food sources, are well-known for their medicinal qualities that go beyond their fundamental nutritional roles. Despite having different definitions, legal contexts, and intended applications, the phrases nutraceuticals, dietary supplements, and functional foods are sometimes used interchangeably. To appreciate their responsibilities in therapeutic and preventative healthcare, it is imperative to comprehend these distinctions. The main differences between these categories are shown in the Table 1 below and Table 2 outlines major categories of nutraceuticals, describing their key health benefits and typical sources, with supporting references for each.

**Table 1:** Comparison of nutraceuticals, dietary supplements, and functional foods.

Aspect	Nutraceuticals	Dietary supplements	Functional foods
Definition	Coined by Stephen DeFelice to describe foods with health benefits, the term nutraceutical is now more accurately used for bioactive compounds extracted from foods and delivered in pharmaceutical forms like capsules or tablets for therapeutic use (76, 77)	While the FDA defines dietary supplements as products intended to supplement the diet (69), many are labeled this way due to the lack of a regulatory category for “nutraceuticals” in countries like the U. S., despite having therapeutic or preventive functions.	Foods that offer benefits beyond basic nutrition when consumed as part of the daily diet (78).
Form	Found as capsules, powders, functional beverages, or herbal extracts (79).	Commonly in tablets, capsules, soft gels, powders, or liquids (69).	Delivered in the form of regular foods like fortified juices, cereals, and probiotic yogurts (Martirosyan et al., 2015).
Purpose	Designed to prevent or treat chronic conditions like diabetes, CVD, arthritis (76).	Intended to meet nutritional needs and support specific health functions like bone health, immunity, etc. (80).	Aim to reduce disease risk or enhance physiological performance (79).
Regulation	Often lacks uniform global regulation; may fall under “food-drug” interface products (76).	Regulated as food, not drugs; labeling must not claim to diagnose or cure diseases (69).	Regulated as conventional foods; health claims must be substantiated (Martirosyan et al., 2015).
Examples	Curcumin, resveratrol, glucosamine, omega-3 fatty acids (79).	Vitamin D, fish oil capsules, folic acid, calcium tablets (69).	Probiotic yogurt, oats with added beta-glucan, vitamin-D fortified milk (Martirosyan et al., 2015).
Scientific Basis	Requires preclinical and clinical studies to support efficacy and safety claims (3).	Evidence may be based on known nutrient functions; less rigorous than pharmaceuticals (76).	Must demonstrate a functional effect (e.g., cholesterol-lowering, gut health improvement) through scientific trials (Martirosyan et al., 2015).

Many of these compounds may fall under more than one category. For example, nutraceuticals are often classified as dietary supplements due to regulatory limitations. Dosage, form, and intended use can influence classification.

**Table 2:** Categories of nutraceuticals, benefits, and sources.

Category	Description	Health benefits	Examples	Type of evidence	Citation
Dietary Fibers	Indigestible plant-based carbohydrates that promote gut health and metabolic regulation.	Lowers cholesterol, regulates blood sugar, supports digestion.	Psyllium, inulin, beta-glucan	Clinical trials, Systematic reviews	Slavin (81) and Khalid et al. (82)
Probiotics and Prebiotics	Probiotics are beneficial live microbes; prebiotics are substrates (not limited to fibers) that selectively promote their growth and activity in the gut.	Improve gut microbiota, enhance immunity, reduce IBS and inflammation.	*Lactobacillus*, FOS, GOS	Clinical trials, Systematic reviews	Gibson et al. (83) and Ji et al. (84)
Polyunsaturated Fatty Acids (PUFAs)	Essential fatty acids with structural and signaling roles in cells.	Anti-inflammatory, cardioprotective, neuroprotective.	Omega-3 (EPA, DHA), omega-6	(DHA), omega-6 Clinical trials, Animal studies	Calder (85) and Mititelu et al. (86)
Polyphenols	Bioactive plant compounds with strong antioxidant and anti-inflammatory effects.	Prevent oxidative stress, reduce cancer risk, support brain and heart health.	Curcumin, resveratrol, catechins	In vitro, Animal studies, Clinical trials	Pandey and Rizvi (87) and Rudrapal et al. (88)
Vitamins and Minerals	Micronutrients essential for immunity, cellular functions, and metabolic health.	Boost immune function, aid in antioxidant defense, support bones and nervous system.	Vitamins A, C, D, iron, zinc	Clinical trials, Systematic reviews	Gombart et al. (89) and Mitra et al. (90)

Bioactive substances called nutraceuticals come from a variety of natural sources, including as microbes, plants, and marine life. The most researched and commonly utilized nutraceuticals are those produced from plants, which include substances like terpenoids, polyphenols, flavonoids, and alkaloids. These phytochemicals have strong anti-inflammatory, antioxidant, and cardioprotective qualities and are widely distributed in fruits, vegetables, herbs, spices, and whole grains (4). The abundance of omega-3 fatty acids (EPA and DHA), carotenoids like astaxanthin, polysaccharides, and bioactive peptides from fish, algae, and shellfish has led to a rise in interest in marine-derived nutraceuticals. According to Shahidi et al. (5), these substances have been connected to anti-cancer, cognitive function support, and cardiovascular advantages. Probiotics and fermentation metabolites are examples of microbial-derived nutraceuticals that have demonstrated effectiveness in regulating gut microbiota, boosting immunological response, and promoting gastrointestinal health. Usually, they come from strains of *Bifidobacterium* and *Lactobacillus*, which are frequently found in fermented foods like kimchi and yogurt (6).

**Figure 1:**
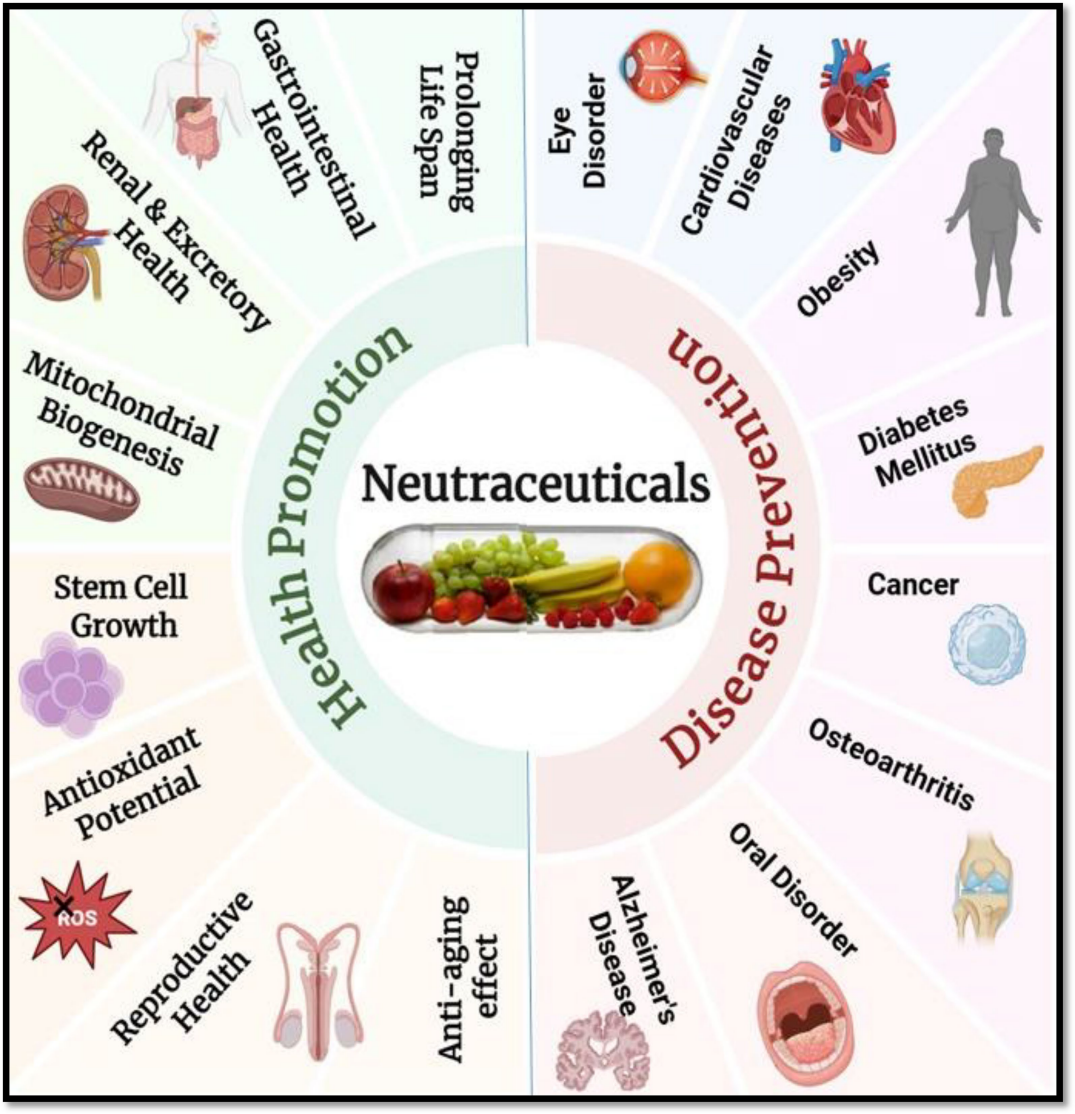
Multifaceted benefits of nutraceuticals in health promotion and disease prevention. Created with BioRender.com.

Nutraceuticals are essential to preventative healthcare because they help to preserve physiological balance and lower the risk of chronic diseases before they manifest. For example, by altering metabolic pathways and lowering oxidative stress, consistent consumption of dietary fibers and polyphenols can prevent diseases including type 2 diabetes, hypertension, and obesity (7). By focusing on disease-specific molecular pathways, these substances aid in or improve treatment results in therapeutic healthcare. By causing apoptosis and preventing tumor growth, nutraceuticals such as curcumin and resveratrol have demonstrated promise as adjuvant cancer treatments (8). Therefore, including nutraceuticals into dietary and medical procedures presents a viable, low-toxicity, and multipurpose strategy for managing chronic illnesses and enhancing general health (Figure 1). Illustrates the extensive benefits of nutraceuticals by categorizing their impact into two major areas: health promotion and disease prevention. It represents how nutraceuticals bioactive compounds derived from food sources, support various physiological functions and help maintain overall wellness. On the health promotion side, the figure highlights how nutraceuticals contribute to gastrointestinal and renal health, mitochondrial biogenesis, stem cell growth, antioxidant activity, reproductive health, anti-aging effects, and lifespan extension. These benefits demonstrate their role in enhancing bodily functions and preventing premature aging. On the disease prevention side, the figure showcases how nutraceuticals help protect against and manage several chronic diseases, including cardiovascular disorders, obesity, diabetes mellitus, cancer, osteoarthritis, Alzheimer’s disease, oral disorders, and eye conditions. By incorporating nutraceuticals into the diet, individuals may improve their health, boost their body’s natural defenses, and reduce the likelihood of disease development.

## 5 Mechanisms of action

**Figure 2:**
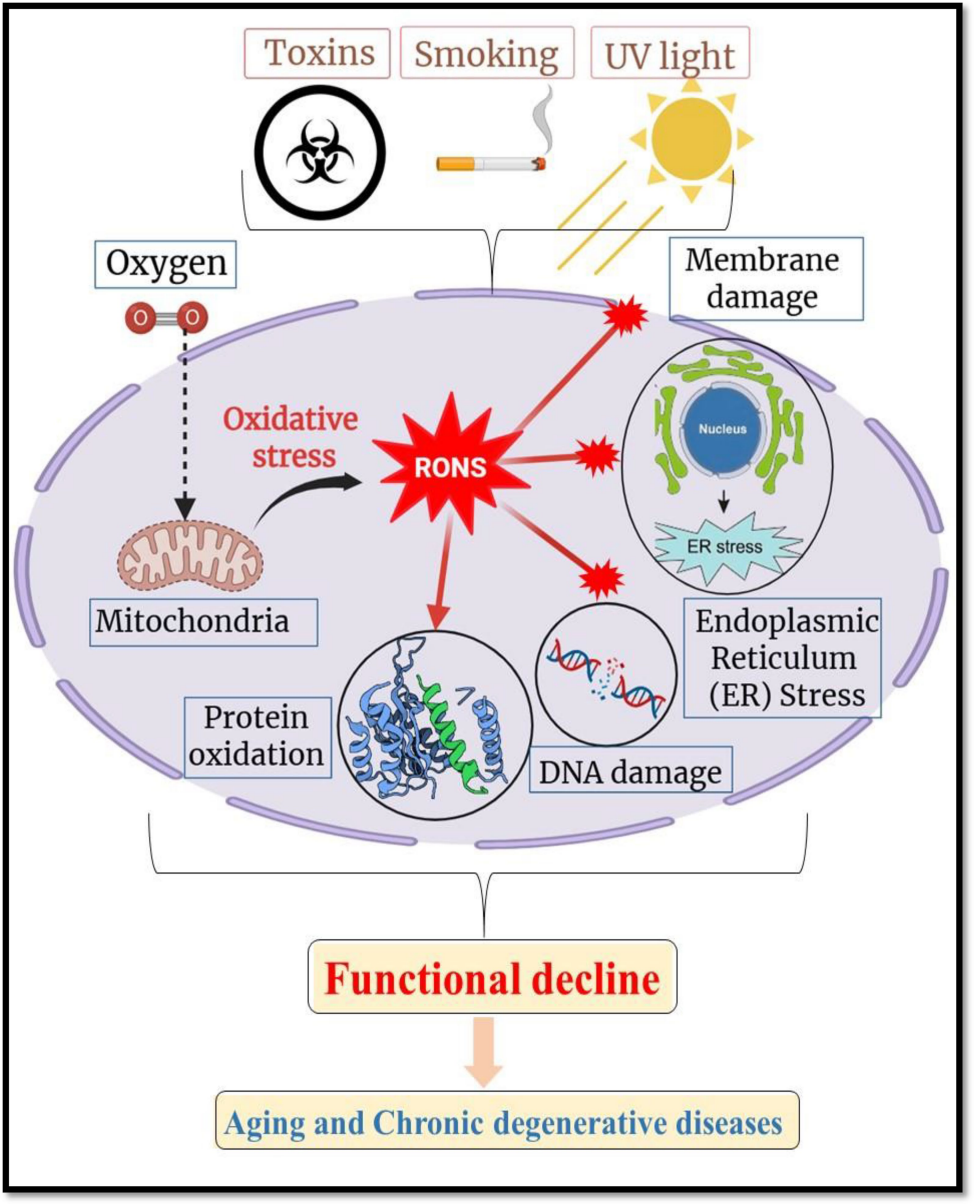
Schematic illustration of oxidative stress and RONS-mediated cellular damage in aging and degenerative diseases. Created with BioRender.com.

Numerous biological processes, many of which interact with crucial pathways implicated in the development and progression of chronic diseases, allow nutraceuticals to provide their therapeutic advantages. Their antioxidant activity is one of the main processes; substances like polyphenols, carotenoids, and vitamins (like C and E) neutralize reactive oxygen species (ROS), shielding cells from damage brought on by oxidative stress. According to Pisoschi et al. (9), oxidative stress has a significant role in aging and a number of chronic illnesses, including cancer, neurodegeneration, and cardiovascular disease (Figure 2). Presents a schematic representation of oxidative stress and its pivotal role in cellular damage leading to aging and chronic degenerative diseases. It highlights how reactive oxygen and nitrogen species (RONS) produced endogenously or triggered by external factors such as toxins, smoking, and ultraviolet (UV) light, accumulate within cells, inducing widespread oxidative stress. This oxidative burden causes multiple forms of molecular and cellular damage, including: DNA damage, which can lead to mutations and genomic instability; Protein oxidation, impairing structural and enzymatic functions; Membrane damage, disrupting cellular compartmentalization and signaling; Mitochondrial dysfunction, diminishing energy production and amplifying ROS generation; Endoplasmic reticulum (ER) stress, which contributes to protein misfolding and cell stress responses (10). In order to maintain cellular homeostasis, polyphenols like resveratrol and flavonoids like quercetin scavenge free radicals and increase the activity of endogenous antioxidant enzymes like glutathione peroxidase and superoxide dismutase. The anti-inflammatory properties of nutraceuticals are another important mechanism. The pathophysiology of conditions including cancer, diabetes, and arthritis is linked to chronic inflammation. Nutraceuticals like gingerols, curcumin, and omega-3 fatty acids block important inflammatory enzymes like COX-2 and iNOS and limit the expression of pro-inflammatory cytokines like TNF-*α* and IL-6 (11). Additionally, these substances suppress transcription factors such as NF-κB, which is essential for fostering immunological dysregulation and inflammation (12).

Another crucial component of nutraceutical function is immune response modulation. By altering dendritic cell activity, boosting T and B lymphocyte proliferation, and encouraging immunoglobulin production, certain probiotics, vitamins (such as vitamin D), and polysaccharides from mushrooms and algae improve both innate and adaptive immune responses (13). Beta-glucans, for example, have been shown to excite natural killer (NK) cells and macrophages, enhancing host defense against cancers and infections (14). Nutraceuticals also affect epigenetic changes including DNA methylation, histone acetylation, and miRNA expression, as well as the control of gene expression. Because they can change the expression of genes involved in cell proliferation, apoptosis, and detoxification, nutrients such as sulforaphane, genistein, and folate are intriguing candidates for epigenetic therapy, particularly in the prevention of cancer (15).

Additionally, there is mounting evidence that nutraceuticals play a part in the regulation of gut microbiota. While inhibiting pathogenic species, prebiotics like inulin and polyphenols like catechins encourage the growth of good gut bacteria like *Bifidobacterium* and *Lactobacillus*. According to Ojeda et al. (16), this microbial balance promotes metabolic health, lowers systemic inflammation, and strengthens the intestinal barrier. Lastly, a lot of nutraceuticals have an impact on signal transduction pathways that control growth, metabolism, inflammation, and cell survival. For instance, resveratrol and curcumin have anti-inflammatory, anti-proliferative, and insulin-sensitizing properties by modulating pathways such NF-κB, MAPK, AMPK, and PI3K/Akt (17, 18). Nutraceuticals’ tailored modulation of these pathways, which are frequently dysregulated in chronic illnesses, provides a multifaceted treatment strategy with low toxicity.

## 6 Role of nutraceuticals in specific chronic diseases

### 6.1 Cardiovascular diseases (CVD)

The top cause of death worldwide is still cardiovascular diseases (CVD), which include hypertension, atherosclerosis, myocardial infarction, and stroke (19). The pathophysiology of CVD is greatly influenced by lifestyle variables such chronic inflammation, poor nutrition, and inactivity (20). By focusing on important risk factors like dyslipidemia, hypertension, and endothelial dysfunction, nutraceuticals present intriguing alternatives to traditional treatments. The cardioprotective benefits of omega-3 fatty acids, especially eicosapentaenoic acid (EPA) and docosahexaenoic acid (DHA), which are mostly found in fish oils, are well established (21). They assist in lowering blood pressure, improving vascular function, inhibiting platelet aggregation, and lowering plasma triglyceride levels (22). By lowering oxidative stress indicators in vascular tissues and downregulating pro-inflammatory cytokines like IL-6 and TNF-*α*, omega-3 PUFAs have anti-inflammatory effects (23). These outcomes help to stabilize pre-existing atherosclerotic plaques and prevent new ones from forming.

**Figure 3:**
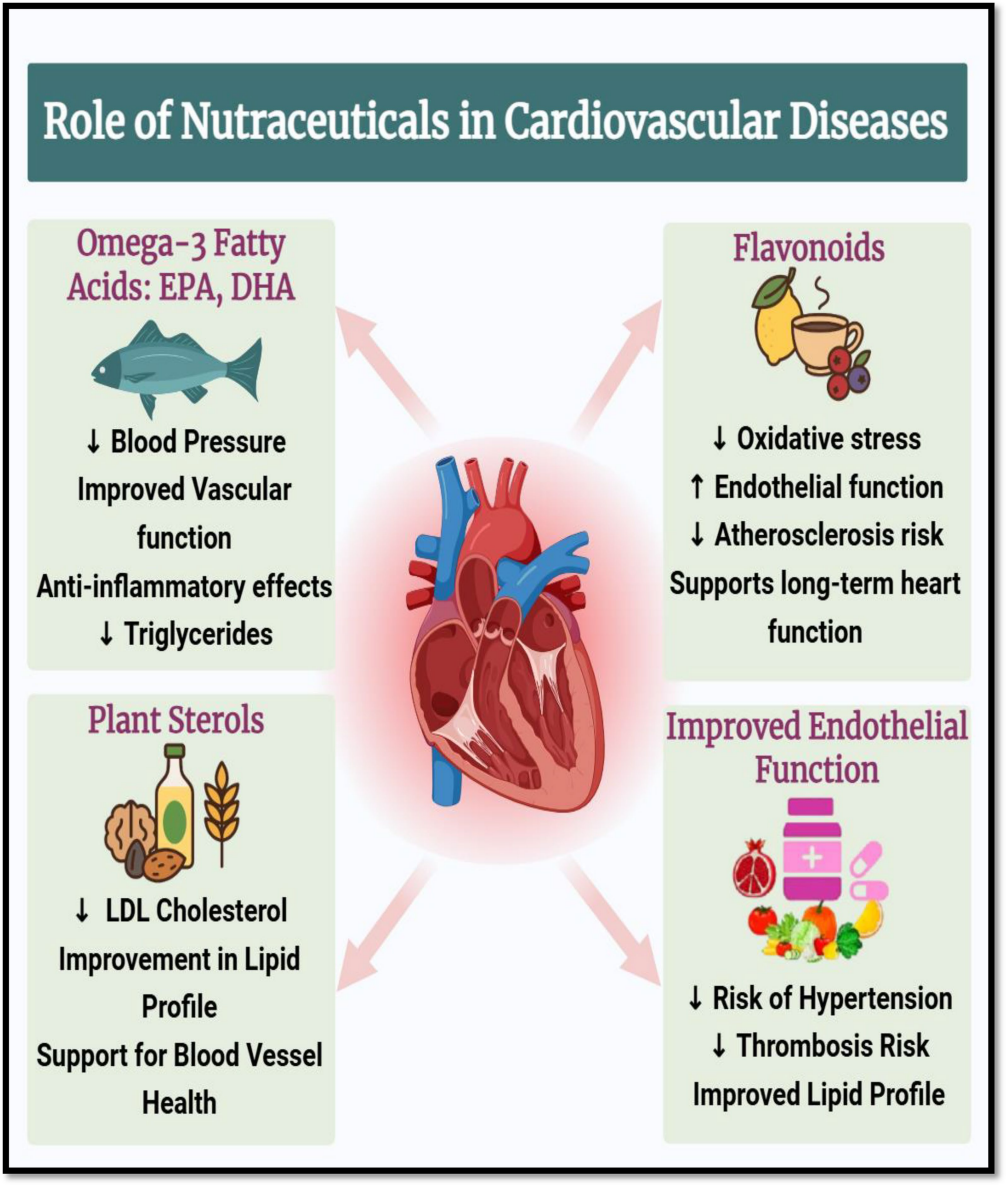
Cardioprotective roles of nutraceuticals in cardiovascular diseases. Created with BioRender.com.

Strong antioxidants and vasodilators, flavonoids are a type of polyphenolic substances that can be found in citrus fruits, tea, berries, and cocoa. By lowering oxidative stress in vascular tissues and raising nitric oxide (NO) bioavailability, they enhance endothelial function (24). Diets high in flavonoids have been linked to improved lipid profiles and decreased blood pressure, especially through improvements in HDL cholesterol and decreases in LDL cholesterol (25). They also alter lipid metabolism-related enzyme systems like lipoprotein lipase and HMG-CoA reductase. Naturally found in nuts, seeds, and vegetable oils, plant sterols and stanols structurally resemble cholesterol and competitively prevent the intestinal absorption of it. Without influencing HDL or triglyceride levels, this lowers serum LDL cholesterol levels (25). When paired with other lifestyle changes, a daily consumption of 2 grams of plant sterols can reduce cardiovascular risk and LDL cholesterol by up to 10% (26). When taken as a whole, these nutraceuticals help to improve endothelial function, blood pressure regulation, and lipid profiles three key areas in the prevention and treatment of CVD. They are useful in integrative cardiometabolic healthcare because their incorporation into dietary plans offers a low-toxicity, natural method of lowering cardiovascular risk (Figure 3). Illustrates the positive effects of nutraceuticals in the prevention and management of cardiovascular diseases (CVD). It highlights the cardio protective roles of omega-3 fatty acids (EPA and DHA), flavonoids, and plant sterols, each acting on key risk factors associated with CVD, including dyslipidemia, endothelial dysfunction, inflammation, and hypertension (26).

### 6.2 Diabetes mellitus

The chronic metabolic disease known as diabetes mellitus, and more specifically type 2 diabetes (T2DM), is typified by hyperglycemia, insulin resistance, and decreased insulin secretion. The prevalence of diabetes is rising worldwide, which emphasizes the need for complementary therapy in addition to traditional medications. Nutraceuticals have great therapeutic potential for controlling blood sugar levels and preserving pancreatic function because of their capacity to alter several metabolic pathways with few adverse effects. Traditional medicine has long utilized bitter melon (*Momordica charantia*) for its hypoglycemic properties. It contains bioactive substances including vicine, polypeptide-p, and charantin that act in an insulin-like manner, enhance glucose uptake in peripheral tissues, and reduce intestinal glucose absorption (27). Experimental studies further show that bitter melon alters key carbohydrate-metabolizing enzymes and promotes pancreatic *β*-cell regeneration (28), while both animal and clinical trials demonstrate its ability to lower fasting blood glucose and HbA1c levels.

By stimulating GLUT4 translocation in adipose and muscle tissues and activating insulin receptor kinase, cinnamon (*Cinnamomum* spp.), particularly its polyphenolic component cinnamaldehyde, improves insulin sensitivity and glucose absorption (29). Evidence from both experimental and human studies indicates that cinnamon also reduces postprandial glucose spikes by inhibiting intestinal *α*-glucosidase and modulating glycogen synthase (30). In addition, its antioxidant properties may help mitigate oxidative stress associated with diabetic complications. Similarly, berberine, an alkaloid isolated from plants such as *Berberis aristata*, has shown antidiabetic effects comparable to metformin. Preclinical studies demonstrate its ability to enhance glucose utilization, inhibit hepatic gluconeogenesis, and increase insulin sensitivity via activation of the AMP-activated protein kinase (AMPK) pathway (31). Clinical evidence further supports its role in improving lipid metabolism and protecting pancreatic *β*-cells in diabetic patients (32).

The metabolism of fats and carbohydrates also depends heavily on chromium, an essential trace metal (33). In individuals with chromium deficiency, supplementation with chromium picolinate has been shown in clinical trials to enhance insulin receptor activation, improve glucose tolerance, and reduce insulin resistance (34). By improving insulin signaling, chromium facilitates cellular glucose uptake and may reduce oxidative stress in diabetes. Collectively, these nutraceuticals—through mechanisms supported by both preclinical and clinical studies—help preserve pancreatic β-cell function, improve insulin sensitivity, and regulate glucose metabolism, making them promising candidates for integrative approaches in type 2 diabetes prevention and management. Compared to synthetic antidiabetic medications, their inclusion in dietary or supplement regimens may provide supportive glycemic control with fewer side effects (35) (Table 3). highlights key nutraceuticals used in managing T2DM, detailing their active compounds, mechanisms of action, and observed effects on blood glucose control and insulin sensitivity, supported by relevant experimental and clinical evidence.

**Table 3:** Nutraceuticals in the management of type 2 diabetes mellitus (T2DM).

Nutraceutical	Active compounds	Mechanisms of action	Effects on diabetes	References
Bitter Melon (*Momordica charantia*)	Vicine, Polypeptide-p, Charantin	- Mimics insulin action - Enhances glucose uptake in peripheral tissues - Inhibits intestinal glucose absorption - Modulates carbohydrate metabolism enzymes - Promotes β-cell regeneration	- Lowers fasting blood glucose - Reduces HbA1c - Supports pancreatic function	Richter et al. (91) and Saqulain et al. (92)
Cinnamon (Cinnamomum spp.)	Cinnamaldehyde (polyphenol)	- Stimulates GLUT4 translocation - Activates insulin receptor kinase - Inhibits *α*-glucosidase - Modulates glycogen synthase - Provides antioxidant protection	- Improves insulin sensitivity - Reduces postprandial glucose spikes - Mitigates oxidative stress	Qin et al. (93) and Tuzcu et al. (94)
Berberine (from Berberis aristata)	Berberine (alkaloid)	- Activates AMPK pathway - Enhances glucose uptake - Inhibits hepatic gluconeogenesis - Increases insulin sensitivity - Improves lipid metabolism - Protects *β*-cells	- Comparable to metformin - Enhances insulin action - Supports β-cell health	Wang et al. (95) and Zieniuk and Pawełkowicz (96)
Chromium (Chromium picolinate)	Chromium (essential trace element)	- Enhances insulin receptor activation - Improves glucose tolerance - Decreases insulin resistance - Reduces oxidative stress	- Facilitates glucose entry into cells - Improves insulin signaling - Beneficial in chromium-deficient individuals	Hua et al. (97) and Inyang et al. (98)

### 6.3 Cancer

Uncontrolled cell proliferation, apoptosis evasion, prolonged angiogenesis, and metastasis are the hallmarks of cancer, which continues to rank among the world’s top causes of illness and mortality (36). Despite their effectiveness, traditional cancer therapies like radiation and chemotherapy sometimes cause serious side effects and resistance. Because of their low toxicity, multitargeted methods of action, and ability to improve therapeutic outcomes, nutraceuticals present attractive supplementary strategies. Curcumin, resveratrol, epigallocatechin gallate (EGCG), and sulforaphane are among the naturally occurring substances whose anti-cancer effects have been well investigated (37). The main polyphenol in turmeric (*Curcuma longa*), curcumin, has strong anti-cancer properties against a range of cancers. It lowers survival pathways like NF-κB, STAT3, and PI3K/Akt and triggers apoptosis by activating caspases and altering Bcl-2 family proteins. By suppressing VEGF and preventing the growth of endothelial cells, curcumin also has anti-angiogenic properties that reduce tumor vascularization. Additionally, it stops the growth of cancer cells by causing cell cycle arrest at the G2/M phase (38).

Resveratrol, a stilbene polyphenol found in grapes, berries, and peanuts, targets various signaling pathways implicated in carcinogenesis. It suppresses angiogenesis by lowering the production of VEGF and HIF-1α and encourages apoptosis through p53 activation and mitochondrial failure. By altering cyclin-dependent kinases (CDKs) and their inhibitors, resveratrol also causes cell cycle arrest, especially in the S and G1 phases (39, 40). Its antioxidant qualities also lessen oxidative DNA damage, which is a major factor in tumor growth and mutagenesis. The most prevalent catechin in green tea, EGCG (epigallocatechin-3-gallate), exhibits anticancer properties via a variety of pathways. In cancer cells, it inhibits telomerase activity, causes apoptosis, and decreases angiogenesis. Additionally, EGCG suppresses matrix metalloproteinases (MMPs) and inhibits signaling pathways like Akt and MAPK, which lessens tumor dissemination and invasiveness (17, 18). Its chemopreventive potential is further enhanced by its capacity to scavenge free radicals and bind metal ions (41).

**Figure 4:**
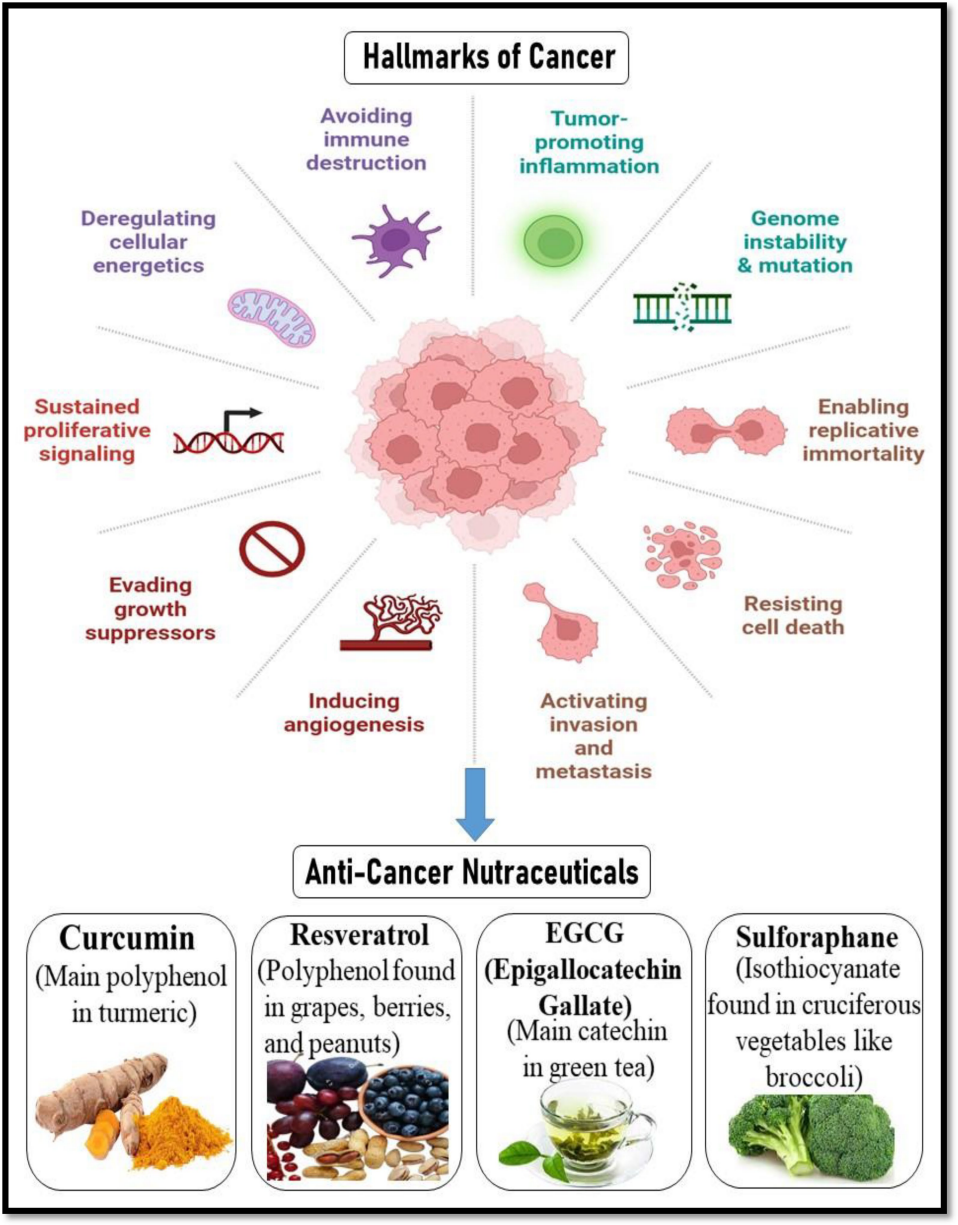
Hallmarks of cancer and the role of anti-cancer nutraceuticals. Created with BioRender.com.

Broccoli and other cruciferous vegetables contain sulforaphane, an isothiocyanate that has been shown to be a potent inducer of phase II detoxification enzymes and a modulator of epigenetic control. It reduces angiogenesis by decreasing VEGF and HIF-1α, causes cell cycle arrest at G2/M, and stimulates apoptosis via mitochondrial mechanisms. Additionally, sulforaphane affects DNA methyltransferases (DNMTs) and histone deacetylases (HDACs), indicating a potential use in epigenetic cancer treatment (42, 43). By causing apoptosis, preventing angiogenesis, and stopping the cell cycle, these nutraceuticals work together to suppress tumor growth and improve the effectiveness of traditional treatments. Their incorporation with chemopreventive measures presents a viable way to lower the risk of cancer and its recurrence (44) (Figure 4). Presents the major hallmarks of cancer, which are fundamental biological traits that allow cancer cells to survive, proliferate, and spread. These hallmarks include sustained proliferative signaling, evading growth suppressors, resisting cell death, enabling replicative immortality, inducing angiogenesis, activating invasion and metastasis, avoiding immune destruction, tumor-promoting inflammation, genome instability and mutation, and deregulating cellular energetics. Together, these capabilities represent how cancer cells bypass normal cellular controls and thrive in the body. The figure also highlights the potential of anti-cancer nutraceuticals- natural compounds such as curcumin, resveratrol, EGCG, and sulforaphane, in targeting these hallmarks. These dietary components, derived from turmeric, grapes, green tea, and cruciferous vegetables respectively, have shown promise in interfering with various mechanisms of cancer progression, offering a complementary strategy for cancer prevention and therapy (45).

### 6.4 Neurodegenerative diseases (Alzheimer’s, Parkinson’s)

Nutraceutical formulations and natural compounds have shown promise in combating shared pathogenic pathways in Alzheimer’s and Parkinson’s diseases including oxidative stress, neuroinflammation, protein aggregation (Aβ, tau, *α*-synuclein), mitochondrial dysfunction, and synaptic impairment. Notably, curcumin though hampered by poor oral bioavailability and rapid clearance exhibits anti-amyloid, antioxidant, and anti-inflammatory effects; advanced nano- and polymeric delivery systems (liposomes, PLGA/lipid nanoparticles, dendrimers, nanomicelles) significantly enhance its gastrointestinal absorption, plasma half-life, brain penetration (up to ~16 × bioavailability), and therapeutic efficacy in preclinical rodent models, and early clinical work in Parkinson’s and Alzheimer’s supports improved delivery and safety profile (46). Green-tea–derived EGCG protects against oxidative and inflammatory neurotoxicity, reduces amyloid and α-synuclein aggregation, preserves dopaminergic neurons in MPTP and 6-OHDA models, and activates the Nrf2/HO-1 pathway (47). Flavonoids like quercetin and apigenin confer neuroprotection *in vitro* and *in vivo* through antioxidant defense, anti-amyloidogenic effects, synaptic support, and neuroinflammation modulation. Resveratrol modulates NF-κB/JNK/MAPK signaling, SIRT1 activation, and microglial cytokine release, offering neuroinflammatory and anti-amyloid benefits—though clinical outcomes remain mixed and oral bioavailability low (~0.5%), prompting exploration of improved formulations (48). Huperzine A, an alkaloid cholinesterase and NMDA-receptor inhibitor, crosses the blood–brain barrier and has shown cognitive-benefit signals in AD trials, despite methodological limitations of existing studies. Complementing these are omega-3 fatty acids, sulfur-rich sulforaphane, carotenoids, and marine phlorotannins, which together address neuroinflammation, mitochondrial health, and proteostasis. While clinical trials (e.g., curcumin, resveratrol, luteolin, crocin, EGb 761) report some cognitive or biomarker improvements in mild AD, they often suffer from low bioavailability, small cohorts, and inconsistent methodologies. Future progress requires standardized, well-powered RCTs, multi-compound synergistic formulations, and advanced CNS-targeted delivery platforms (e.g., nanoparticles, nanomicelles, NADES), alongside robust pharmacokinetics, dosage optimization, gut–brain axis evaluation, and safety characterization to translate these nutraceuticals into effective adjunctive neurotherapeutic regimens (49).

### 6.5 Inflammatory and autoimmune disorders

Dysregulated immune responses, chronic inflammation, and compromised immunological tolerance are the causes of autoimmune and inflammatory diseases, including multiple sclerosis (MS), rheumatoid arthritis (RA), inflammatory bowel disease (IBD), and systemic lupus erythematosus (SLE). Because traditional immunosuppressive treatments are frequently linked to side effects and an elevated risk of infection, there is growing interest in nutraceuticals as safer, complementary alternatives. By controlling cytokines, T-cell activity, and the composition of the gut microbiota, substances such as probiotics, turmeric, ginger, and boswellia have shown immunomodulatory qualities (50).

Strong anti-inflammatory properties are demonstrated by turmeric (*Curcuma longa*), especially its active polyphenol curcumin, which inhibits the expression of pro-inflammatory cytokines like TNF-*α*, IL-1β, and IL-6. One important regulator of inflammation and autoimmunity, the NF-κB signaling pathway, is inhibited. Curcumin also reduces chronic inflammation at the molecular level by downregulating inducible nitric oxide synthase (iNOS) and cyclooxygenase-2 (COX-2). Curcumin has also been demonstrated to alter T-cell differentiation, boosting regulatory T-cells (Tregs) and inhibiting Th17 cells to restore immunological balance (51).

Bioactive substances called gingerols and shogaols, which are found in ginger (*Zingiber officinale*), have been demonstrated to prevent the synthesis of pro-inflammatory mediators such prostaglandins and leukotrienes. Ginger inhibits the activation of NF-κB and lowers the release of inflammatory cytokines by T-cells and macrophages. Due to its inhibition of the COX and lipoxygenase (LOX) pathways, ginger has been shown in clinical tests to help individuals with osteoarthritis and rheumatoid arthritis experience less pain and stiffness (52, 53).

The boswellic acids produced by *Boswellia serrata*, commonly referred to as *Indian frankincense*, target the enzyme 5-lipoxygenase (5-LOX), which is in charge of leukotriene production. In diseases including RA, asthma, and IBD, this activity helps lower inflammation. Boswellic acids can be used to control autoimmune diseases since they also decrease TNF-α and IL-6 and enhance immunological regulation by reducing CD4 + T-cell proliferation and activation (54). By altering the gut microbiota, probiotics—particularly strains like *Lactobacillus* and *Bifidobacterium*—have an impact on immunological responses both locally and systemically. They improve intestinal barrier integrity, lower systemic endotoxin burden, and alter dendritic cell and T-cell responses by reestablishing microbial balance (55). According to Ouwehand et al. (56), probiotics are known to reduce the production of inflammatory cytokines and enhance anti-inflammatory markers such as IL-10, which helps to reduce symptoms of autoimmune illnesses and induce remission in IBD. Together, these nutraceuticals provide a multifaceted and all-natural method of treating inflammatory and autoimmune diseases by regulating T-cells, balancing the microbiota, and modulating cytokines. Their inclusion in treatment plans may help maintain long-term immunological health, decrease side effects, and lessen reliance on synthetic immunosuppressants (57).

## 7 Formulation strategies for enhanced efficacy

The poor bioavailability of nutraceuticals, which is frequently caused by low solubility, instability in the gastrointestinal system, quick metabolism, and poor absorption, is a significant obstacle restricting their clinical application, despite their great therapeutic potential. Advanced formulation techniques have been created to solve these problems and guarantee effective distribution to the intended tissues (58). These include targeted delivery systems, bioavailability enhancers, encapsulating methods, and nano-formulations, all of which are essential for maximizing the effectiveness of nutraceuticals. Liposomes and nanoparticles are examples of nano-formulations that have drawn a lot of interest because of their capacity to increase solubility, shield bioactive substances from deterioration, and promote cellular uptake. The stability and controlled release of nutraceuticals such as curcumin, EGCG, and resveratrol can be improved by liposomes, which are spherical vesicles made of phospholipid bilayers that can encapsulate both hydrophilic and lipophilic substances (59). Likewise, polymeric and lipid-based nanoparticles enhance pharmacokinetic characteristics by extending systemic circulation and improving gastrointestinal absorption. Additionally, these nanosystems offer surface changes that improve therapeutic specificity by enabling targeting of particular regions (60).

Encapsulation methods, such as hydrogel systems and microencapsulation, are frequently used to shield delicate nutraceuticals from gastrointestinal and environmental deterioration. Encasing bioactive substances in microscopic carriers composed of biopolymers as chitosan, gelatin, or alginate is known as microencapsulation. In addition to protecting the substances from oxidation and pH changes, this method enables regulated release and covers up offensive tastes or odors (61). Probiotics and polyphenols have been encapsulated in hydrogels, which are cross-linked, water-swollen polymeric networks that improve their release at specific intestinal locations and guarantee their passage through the digestive tract. A number of methods for improving bioavailability have been developed in order to overcome first-pass metabolism and limited absorption. Piperine co-administration is one of the most well-known, especially when combined with curcumin. According to Shoba et al. (62, 63), piperine, an alkaloid found in black pepper, increases the plasma concentration of curcumin by up to 2000% by blocking intestinal and hepatic glucuronidation. Emulsification, which involves adding the nutraceutical to oil-in-water emulsions, is another technique that enhances the solubility and absorption of lipophilic substances including fat-soluble vitamins and carotenoids (64).

By guiding the active ingredient to the site of action while reducing systemic exposure and adverse effects, targeted delivery systems are also essential for increasing efficacy. Enzyme-sensitive release mechanisms, pH-responsive carriers, or ligand-functionalized nanoparticles can all do this. For example, nutraceuticals can be delivered directly to cancer cells that overexpress folate receptors using folic acid-conjugated nanoparticles (65). By lowering the necessary dosage and improving therapeutic outcomes, such precision techniques improve treatment efficiency and patient satisfaction. All things considered, these formulation techniques greatly enhance the bioavailability, stability, and therapeutic targeting of nutraceuticals, making them more dependable and potent medicines. These methods should soon be further refined by continuing developments in material science and nanotechnology, allowing for disease-specific and customized nutraceutical delivery (Table 4). Summarizes innovative formulation technologies developed to improve the delivery and effectiveness of nutraceuticals. It outlines various strategies such as liposomes, nanotechnology, hydrogels, and self-emulsifying systems, highlighting their associated nutraceuticals, delivery advantages, and key scientific references (66).

**Table 4:** Recent advances in formulation strategies for enhanced nutraceutical delivery.

Formulation strategy	Nutraceuticals (examples)	Key advantages	References
Liposomal Carriers	Vitamin E acetate, Grape seed extract, CoQ10, Melatonin, Resveratrol, Zinc gluconate, Berberine	Improved solubility, stability, bioavailability, skin penetration, and sustained release	Keller (99), Padamwar and Pokharkar (100), Lee and Tsai (101), Amri et al. (102)
Electrospun Fiber Mats	Curcumin, α-Tocopherol, Asiaticoside, Ascorbyl palmitate	Gradual release, ultra-fine mats for topical delivery, improved antibacterial and therapeutic effect	Taepaiboon et al. (103), Suwantong et al. (104, 105), and Paneva et al. (106)
Nano−/Microsponges	Melanin, Resveratrol, Acemannan, Curcumin	Enhanced stability, solubility, tissue regeneration, and skin protection	Patravale and Mandawgade (107), Ansari et al. (108), and Chantarawaratit et al. (109)
Cyclodextrin Complexes	Silymarin, Curcumin, Garlic oil, Vitamin A, Kaempferia parviflora, Berberine	Increased solubility, improved permeability, controlled release	Tonnesen et al. (110), Ghosh et al. (111), Kim et al. (112), and Mekjaruskul et al. (113)
Biodegradable Hydrogels	Riboflavin, *Aloe vera*, Chondroitin sulfate, Probiotics (encapsulation)	Gastric protection, pH-responsive release, improved probiotic viability, sustained delivery	Maltais et al. (114), Elzoghby et al. (115), Piai et al. (116), and Pereira et al. (117)
Nanotechnology	Lutein, CoQ10, Quercetin, EGCG, β-Carotene, Curcumin	Improved absorption, stability, bioaccessibility, and targeted delivery	Mitri et al. (118), Teeranachaideekul et al. (119), Tan et al. (120), and Shpigelman et al. (121)
Solid Dispersions	Vitamin A palmitate, CoQ10, Resveratrol, Curcumin	Solubility enhancement, dissolution improvement, timed release	Patravale (107), Nepal et al. (122), and Weigel et al. (123)
Self-Emulsifying Systems	Milk thistle, Kaempferia parviflora, Curcumin	Rapid absorption, improved bioavailability, lymphatic delivery	Onoue et al. (135), Losio et al. (136), and Mekjaruskul et al. (113)
Microparticles/Microcapsules	Lycopene, Vitamin A, Vitamin E, Probiotics	Prolonged release, better shelf-life, improved viability and masking properties	Rocha et al. (124), Martinez-Sancho et al. (125)
Particle Coatings	CoQ10, Vitamin E, Probiotics	Enhanced stability, targeted release in the gut, sustained therapeutic action	Zhang (126), and Peng et al. (127)
Nutraceutical Derivatives	EGCG, Vitamin E, Curcumin derivatives	Enhanced antioxidant, anti-inflammatory, and anticancer activity	Zhong et al. (128), Chen et al. (129), and Zhang et al. (130)

## 8 Safety, toxicity, and regulatory aspects

Although nutraceuticals are frequently promoted as natural and safe substitutes for medicines, issues with their toxicity, safety, and regulatory monitoring still need to be addressed. The widespread belief that “natural equals safe” is untrue because using nutraceuticals excessively or inappropriately can have negative effects, particularly in susceptible groups or when taken with concomitant drugs. Integrating nutraceuticals into traditional healthcare systems requires ensuring safe dosing, avoiding negative interactions, and following established laws. The main causes of toxicological issues are incorrect dosage, contamination, adulteration, or extended use without monitoring. Even though a lot of nutraceuticals come from food, their bioactive ingredients might have pharmacological effects at greater dosages when they are condensed into supplements. For example, too much consumption of fat-soluble vitamins (A, D, E, and K) can accumulate in bodily tissues and cause toxicity. Likewise, in sensitive people, elevated levels of green tea catechins have been linked to liver damage (67). Therefore, it is crucial to establish the safe dosage range for each molecule, which needs to be backed up by toxicological research, including LD50 and NOAEL (No Observed Adverse Effect Level) data.

The possibility of herb-drug interactions, in which specific nutraceuticals may change the pharmacokinetics or pharmacodynamics of prescription medications, is a serious safety concern. St. John’s Wort, for instance, can increase cytochrome P450 enzymes, particularly CYP3A4, which decreases the effectiveness of medications including immunosuppressants, anticoagulants, and oral contraceptives. Similarly, when combined with anticoagulants like warfarin, substances like ginseng, garlic, and *ginkgo biloba* may raise the risk of bleeding (68). When mixing nutraceuticals with pharmaceuticals, rigorous screening and clinical guidance are necessary because such interactions may lead to heightened toxicity or therapeutic failure. The legal systems that regulate nutraceuticals differ greatly across the globe. According to the Dietary Supplement Health and Education Act (134), the U.S. Food and Drug Administration (69) (FDA) in the US regulates nutraceuticals as dietary supplements. Although pre-market approval is not necessary for these products, they must refrain from making promises about curing diseases unless they are backed up by proof. Under the Nutrition and Health Claims Regulation (Regulation EC No. 1924/2006), the European Food Safety Authority (EFSA) in the European Union assesses health claims for functional foods and supplements. In order to promote Ayurvedic-based nutraceuticals with standards for quality and labeling, the AYUSH Ministry and FSSAI, respectively, regulate traditional and health supplements in India. Nonetheless, disparities in classification, labeling requirements, and enforcement practices among nations frequently lead to market misunderstandings and a range of product quality (70).

Furthermore, to guarantee efficacy and reproducibility, clinical validation and standardization of nutraceuticals are necessary. Many products lack thorough human clinical trials and are instead promoted on the basis of preclinical or anecdotal evidence. Different brands and batches may experience different results because to variations in the sourcing of raw materials, extraction techniques, and concentrations of active compounds. Consequently, quality assurance, standardization of active substances, and compliance with Good Manufacturing Practices (GMP) are essential. To assess the clinical efficacy and safety profile of nutraceuticals across populations and disease situations, long-term randomized controlled studies (RCTs) are also required (71). To support clinical translation, the most commonly studied nutraceutical bioactive compounds, their typical dosages (posology), and reported adverse effects are summarized in Table 5. This table provides a quick reference for clinicians and researchers, highlighting evidence-based dosage ranges from clinical studies and known safety concerns, thereby serving as a practical tool for integrating nutraceuticals into clinical practice.

**Table 5:** Common nutraceutical bioactive compounds with typical dosages and reported adverse effects (based on clinical and preclinical evidence).

Nutraceutical/bioactive compound	Typical dosage range (from clinical studies)	Reported adverse effects	References
Curcumin (*Curcuma longa*)	500–2000 mg/day, often with piperine to enhance bioavailability	Mild gastrointestinal discomfort, nausea, risk of bleeding at high doses	Shoba et al. (62, 63), Shanmugam et al. (38), Afshari et al. (8)
Resveratrol (grapes, berries, peanuts)	150–1,000 mg/day	Headache, GI upset; high doses may affect liver enzymes	Athar et al. (40), Amri et al. (102), and Ansari et al. (108)
Epigallocatechin gallate (EGCG, green tea catechin)	300–8;00 mg/day	Rare hepatotoxicity at high doses, insomnia, GI upset	Singh et al. (131), Mazzanti et al. (67), and Zhong et al. (128)
Omega-3 fatty acids (EPA/DHA, fish oil)	1–4 g/day	Fishy aftertaste, GI upset, increased bleeding risk at high intake	Mozaffarian and Wu (23), Calder (85), and Bhatt et al. (72)
Berberine (*Berberis* spp.)	500–1,500 mg/day	Constipation, cramps, potential CYP450 interactions	Yin et al. (31) and Wang et al. (95)
Cinnamon (*Cinnamomum* spp.)	1–6 g/day bark powder or 250–500 mg extract/day	Allergic reactions, potential hepatotoxicity (Cassia type due to coumarin)	Khan et al. (30), Qin et al. (93), and Senevirathne et al. (29)
Bitter melon (*Momordica charantia*)	500–2000 mg/day extract (varies by preparation)	Hypoglycemia, GI upset	Hasan As'ari et al. (28), Goyal et al. (132), and Richter et al. (91)
Quercetin (flavonoid)	500–1,000 mg/day	Headaches, tingling; kidney concerns at very high doses	Tan et al. (120) and Singh et al. (17, 18)
Boswellia (Boswellic acids)	300–600 mg extract, 2–3 × daily	GI upset, nausea, rare liver enzyme elevation	Siddiqui (54) and Daily et al. (52, 53)
Probiotics (*Lactobacillus*, *Bifidobacterium*)	10^8^–10^11^ CFU/day	Mild bloating, gas; generally safe	Markowiak and Śliżewska (6), Ouwehand et al. (56), and Ji et al. (84)
Chromium picolinate	200–1,000 μg/day	Headache, sleep disturbance; rare renal/hepatic concerns	Anderson (34) and Hua et al. (97)

## 9 Clinical evidence and limitations

A growing number of clinical research and meta-analyses examining the effectiveness of nutraceuticals in controlling chronic diseases have been prompted by their therapeutic promise. Numerous of these research back up the usage of nutraceuticals in addition to traditional pharmaceuticals as supplementary therapy. Nevertheless, despite promising results, a number of issues pertaining to study design, standardization, and sample size have hindered the conversion of preclinical outcomes into reliable clinical success. Nutraceuticals have been shown to be useful in improving clinical outcomes in a number of significant clinical trials. For example, omega-3 fatty acids significantly decreased cardiovascular events in high-risk patients with hypertriglyceridemia, according to a double-blind, placebo-controlled research (72). In a similar vein, curcumin has demonstrated promise in reducing the symptoms of inflammatory bowel disease and rheumatoid arthritis; meta-analyses have showed decreases in inflammatory markers, disease activity, and pain levels when compared to a placebo (52, 53). In a different randomized study, berberine was found to be just as successful as metformin at lowering blood glucose levels in people with type 2 diabetes (31). When compared to conventional pharmaceutical therapies, these investigations highlight the therapeutic equivalency or synergistic impact of specific nutraceuticals.

However, because to variations in research length, formulation types, and patient groups, efficacy results from different trials continue to be inconsistent, particularly when compared to conventional medications. Even if some studies show notable improvements, others are unable to reproduce these advantages in demanding clinical settings. The clinical significance of several nutraceuticals is called into question by placebo-controlled studies that frequently yield moderate or statistically insignificant outcomes (73). Sometimes, rather than hard clinical outcomes like mortality or illness remission, positive outcomes are restricted to surrogate endpoints (such biomarker alterations). The diversity of clinical research presents a significant obstacle. Because of the considerable variations in dosage, duration, administration methods, and outcome measures found in nutraceutical trials, it is challenging to perform meta-analyses and reach generalizable findings. For instance, various studies may utilize different amounts of EGCG in their green tea extracts, which could result in uneven therapeutic benefits. Furthermore, a lot of trials are underpowered because of their short durations or small sample numbers, which restricts their statistical reliability and generalizability to larger populations (74). Another crucial problem is variation in the bioactive content. Nutraceuticals, in contrast to pharmaceutical medications, are frequently made from entire plants or blended extracts, where the concentration and makeup of active ingredients might vary depending on the region of origin, the cultivation practices, and the extraction techniques used. Even the same product may produce varying findings in different experiments if sufficient standardization is not implemented. In addition to impeding repeatability, this inconsistency makes safety assessments and dose–response analyses more difficult (75).

## 10 Future prospects and research directions

Nutraceuticals have a bright future in healthcare thanks to developments in integrated medicine, tailored nutrition, and biotechnology. Nutraceuticals are anticipated to become increasingly important in reducing the burden of chronic diseases as worldwide awareness of preventative health increases. The creation of individualized nutraceutical regimens based on a person’s genetic composition, microbiome composition, lifestyle, and disease risk profile is one of the main directions. With the help of nutrigenomics and metabolomics, this strategy seeks to maximize therapeutic benefits while reducing side effects. Furthermore, advancements in delivery methods including biosensors, controlled-release platforms, and smart nanocarriers will improve the stability, bioavailability, and focused action of nutraceutical substances. Particularly for substances with limited solubility or fast metabolism, these technologies are probably going to increase patient compliance and efficacy. Combining many nutraceuticals or integrating them with traditional medications in synergistic formulations has the potential to increase therapeutic advantages and more comprehensively treat multifactorial disorders like diabetes, cancer, and neurodegeneration.

Furthermore, by forecasting compound-disease connections, evaluating clinical data, and determining ideal dosage ranges, the incorporation of artificial intelligence (AI) and big data analytics in nutraceutical research helps speed up discovery. In order to increase worldwide acceptance, guarantee safety, and encourage the evidence-based use of nutraceuticals, regulatory harmonization and quality standardization among nations will also be essential. Large-scale, carefully planned clinical trials must be given top priority in future studies, along with the investigation of new biomarkers for efficacy and the expansion of our knowledge of long-term impacts, mechanisms of action, and population-specific reactions. Nutraceuticals are positioned to become a crucial component of future healthcare strategies as consumer desire and scientific data align, bridging the gap between medicine and nutrition in the search for preventive, tailored, and sustainable health solutions.

## 11 Conclusion

Through processes like antioxidant activity, inflammatory control, immunological modulation, and metabolic regulation, nutraceuticals and natural substances have become invaluable partners in the prevention and management of chronic diseases. These bioactive substances, which come from a variety of sources such as plants, marine life, and microorganisms, have shown promise in treating complicated illnesses like diabetes, cancer, heart disease, neurological disorders, and autoimmune diseases. Their clinical usefulness and bioavailability have been further improved by advanced formulation techniques such encapsulation technologies and nano-delivery systems. The broad use of nutraceuticals in conventional medicine is hampered by issues with safety, standardization, herb-drug interactions, and inconsistent regulations, despite encouraging preclinical and clinical data. It is still essential to conduct thorough, extensive human studies for clinical validation in order to prove their effectiveness and guarantee reliable treatment results. The future of nutraceutical use will be significantly shaped by the combination of global regulatory frameworks, AI-driven discovery, and customized nutrition.

## Glossary

AI: Artificial Intelligence
AMPK: Activated Protein Kinase
CDK: Cyclin-Dependent Kinase
COX-2: Cyclooxygenase-2
CVD: Cardiovascular Disease
DHA: Docosahexaenoic Acid
DNMT: DNA Methyltransferase
DSHEA: Dietary Supplement Health and Education Act
EGCG: Epigallocatechin-3-Gallate
EPA: Eicosapentaenoic Acid
EFSA: European Food Safety Authority
FDA: Food and Drug Administration
FOS: Fructooligosaccharides
GOS: Galactooligosaccharides
HDAC: Histone Deacetylase
HIF-1*α*: Hypoxia-Inducible Factor 1-alpha
IBD: Inflammatory Bowel Disease
IL: Interleukin
iNOS: Inducible Nitric Oxide Synthase
LOX: Lipoxygenase
MAPK: Mitogen Activated Protein Kinase
MMP: Matrix Metalloproteinase
MS: Multiple Sclerosis
NF-κB: Nuclear Factor Kappa-Light-Chain-Enhancer of Activated B Cells
NK cells: Natural Killer Cells
NCD: Non-Communicable Disease
NOAEL: No Observed Adverse Effect Level
NSAIDs: Nonsteroidal Anti-Inflammatory Drugs
PUFAs: Polyunsaturated Fatty Acids
PI3K/Akt: Phosphoinositide 3-Kinase/Protein Kinase B Pathway
RCT: Randomized Controlled Trial
RA: Rheumatoid Arthritis
ROS: Reactive Oxygen Species
SLE: Systemic Lupus Erythematosus
STAT3: Signal Transducer and Activator of Transcription 3
Th17: T Helper 17 Cells
T2DM: Type 2 Diabetes Mellitus
TNF-α: Tumor Necrosis Factor-alpha
Tregs: Regulatory T Cells
VEGF: Vascular Endothelial Growth Factor
